# Supplementary material for: The impact of digital financial development on corporate leverage ratio: The case of a-share listed non-financial enterprises in China’s Shanghai and Shenzhen stock exchanges
Source: PLoS One. 2024 Aug 12;19(8):e0302978. doi: 10.1371/journal.pone.0302978 (PMC11318853; doi:10.1371/journal.pone.0302978)
Supplement: S1 File — (ZIP) [file pone.0302978.s001.zip › S2.docx]

1.1 基准回归

Regression Table

|  | (1) | (2) | (3) | (4) |
| --- | --- | --- | --- | --- |
|  | lev | lev | slev | llev |
| dfi | -0.213*** | -0.137*** | -0.086*** | -0.050*** |
|  | (-17.668) | (-13.661) | (-8.997) | (-9.225) |
| fix |  | 0.084*** | -0.035*** | 0.119*** |
|  |  | (9.938) | (-4.353) | (23.750) |
| roa |  | -0.744*** | -0.593*** | -0.152*** |
|  |  | (-27.311) | (-24.360) | (-14.319) |
| cashflow |  | -0.127*** | -0.037** | -0.089*** |
|  |  | (-7.332) | (-2.181) | (-10.192) |
| growth |  | 0.000*** | 0.000*** | -0.000*** |
|  |  | (14.242) | (23.396) | (-4.117) |
| tobinq |  | 0.003*** | 0.003** | 0.001*** |
|  |  | (2.890) | (2.529) | (3.738) |
| top1 |  | -0.004 | 0.019*** | -0.023*** |
|  |  | (-0.587) | (2.981) | (-6.409) |
| size |  | 0.069*** | 0.040*** | 0.029*** |
|  |  | (63.145) | (37.592) | (56.481) |
| listage |  | 0.030*** | 0.030*** | 0.001 |
|  |  | (19.426) | (20.261) | (0.709) |
| m2 |  | 0.007*** | 0.006*** | 0.002*** |
|  |  | (10.557) | (8.597) | (4.501) |
| gdp |  | 0.003*** | 0.003*** | 0.000 |
|  |  | (3.585) | (3.755) | (0.046) |
| Cons | 1.672*** | -0.479*** | -0.172*** | -0.313*** |
|  | (23.142) | (-7.424) | (-2.789) | (-9.232) |
| industry | No | No | No | No |
| N | 27739 | 27739 | 27739 | 27739 |
| r2_a | 0.2053 | 0.4620 | 0.3226 | 0.4230 |

1.2 稳健性检验

IV-2SLS回归结果

|  | (1) | (2) | (3) | (4) |
| --- | --- | --- | --- | --- |
|  | lev | lev | slev | llev |
| dfi | -0.193*** | -0.142*** | -0.097*** | -0.045*** |
|  | (-13.774) | (-12.246) | (-8.734) | (-6.910) |
| fix |  | 0.060*** | -0.060*** | 0.120*** |
|  |  | (6.107) | (-6.391) | (19.929) |
| roa |  | -0.738*** | -0.588*** | -0.152*** |
|  |  | (-23.253) | (-20.906) | (-11.901) |
| cashflow |  | -0.121*** | -0.022 | -0.097*** |
|  |  | (-5.615) | (-1.080) | (-9.546) |
| growth |  | 0.002** | 0.002** | 0.000 |
|  |  | (1.990) | (2.219) | (0.451) |
| tobinq |  | 0.002** | 0.002* | 0.001** |
|  |  | (2.124) | (1.678) | (2.100) |
| top1 |  | -0.002 | 0.023*** | -0.026*** |
|  |  | (-0.284) | (3.114) | (-5.891) |
| size |  | 0.072*** | 0.042*** | 0.030*** |
|  |  | (65.575) | (39.895) | (47.480) |
| listage |  | 0.027*** | 0.028*** | -0.001 |
|  |  | (12.535) | (13.769) | (-0.824) |
| 时间效应 | 控制 | 控制 | 控制 | 控制 |
| 行业效应 | 控制 | 控制 | 控制 | 控制 |
| N | 19288 | 19288 | 19288 | 19288 |
| r2_a | 0.0058 | 0.3206 | 0.1849 | 0.1922 |

剔除2015年

|  | (1) | (2) | (3) | (4) |
| --- | --- | --- | --- | --- |
|  | lev | lev | slev | llev |
| dfi | -0.212*** | -0.135*** | -0.086*** | -0.048*** |
|  | (-16.750) | (-12.846) | (-8.615) | (-8.435) |
| fix |  | 0.085*** | -0.034*** | 0.119*** |
|  |  | (9.747) | (-3.993) | (22.605) |
| roa |  | -0.736*** | -0.584*** | -0.152*** |
|  |  | (-26.125) | (-23.290) | (-13.695) |
| cashflow |  | -0.130*** | -0.041** | -0.088*** |
|  |  | (-7.258) | (-2.322) | (-9.529) |
| growth |  | 0.000*** | 0.000*** | -0.000*** |
|  |  | (13.343) | (22.393) | (-3.918) |
| tobinq |  | 0.003** | 0.002** | 0.001*** |
|  |  | (2.275) | (1.964) | (3.252) |
| top1 |  | -0.005 | 0.018*** | -0.023*** |
|  |  | (-0.666) | (2.783) | (-6.176) |
| size |  | 0.069*** | 0.039*** | 0.029*** |
|  |  | (60.603) | (36.136) | (54.167) |
| listage |  | 0.031*** | 0.030*** | 0.000 |
|  |  | (18.982) | (19.978) | (0.438) |
| m2 |  | 0.007*** | 0.006*** | 0.002*** |
|  |  | (10.496) | (8.532) | (4.514) |
| gdp |  | 0.003*** | 0.003*** | 0.000 |
|  |  | (3.569) | (3.723) | (0.070) |
| Cons | 1.670*** | -0.473*** | -0.155** | -0.324*** |
|  | (22.043) | (-6.996) | (-2.408) | (-9.138) |
| 时间效应 | 控制 | 控制 | 控制 | 控制 |
| 行业效应 | 控制 | 控制 | 控制 | 控制 |
| N | 25410 | 25410 | 25410 | 25410 |
| r2_a | 0.2032 | 0.4618 | 0.3231 | 0.4196 |

引入滞后项

|  | (1) | (2) | (3) | (4) |
| --- | --- | --- | --- | --- |
|  | lev | lev | slev | llev |
| L.lev | 0.904*** | 0.851*** |  |  |
|  | (256.481) | (190.015) |  |  |
| L.slev |  |  | 0.840*** |  |
|  |  |  | (177.491) |  |
| L.llev |  |  |  | 0.754*** |
|  |  |  |  | (101.674) |
| dfi | -0.018*** | -0.019*** | -0.017*** | -0.008** |
|  | (-3.098) | (-3.494) | (-3.031) | (-2.058) |
| fix |  | -0.018*** | -0.028*** | 0.022*** |
|  |  | (-3.872) | (-5.673) | (6.223) |
| roa |  | -0.374*** | -0.326*** | -0.065*** |
|  |  | (-21.177) | (-20.324) | (-6.629) |
| cashflow |  | -0.052*** | 0.012 | -0.066*** |
|  |  | (-4.207) | (0.932) | (-8.458) |
| growth |  | 0.000 | 0.000 | 0.000*** |
|  |  | (1.453) | (0.452) | (6.113) |
| tobinq |  | 0.001** | 0.001*** | -0.000 |
|  |  | (2.236) | (3.258) | (-0.228) |
| top1 |  | 0.009** | 0.010*** | -0.003 |
|  |  | (2.508) | (2.651) | (-1.356) |
| size |  | 0.013*** | 0.008*** | 0.008*** |
|  |  | (21.873) | (15.332) | (19.506) |
| listage |  | -0.013*** | -0.007*** | -0.005*** |
|  |  | (-14.859) | (-8.549) | (-7.720) |
| m2 |  | 0.002*** | 0.002*** | -0.000 |
|  |  | (5.626) | (6.434) | (-0.286) |
| gdp |  | -0.000 | 0.000 | -0.000 |
|  |  | (-0.354) | (0.289) | (-0.913) |
| Cons | 0.151*** | -0.097*** | -0.034 | -0.096*** |
|  | (4.372) | (-2.996) | (-1.025) | (-3.871) |
| 时间效应 | 控制 | 控制 | 控制 | 控制 |
| 行业效应 | 控制 | 控制 | 控制 | 控制 |
| N | 23337 | 23337 | 23337 | 23337 |
| r2_a | 0.8472 | 0.8680 | 0.8130 | 0.7426 |

分位数回归

|  | 25百分位 | 50百分位 | 75百分位 |
| --- | --- | --- | --- |
|  | (1) | (2) | (3) |
|  | lev | lev | lev |
| dfi | -0.132*** | -0.136*** | -0.141*** |
|  | (-9.20) | (-12.85) | (-10.22) |
| fix | 0.125*** | 0.085*** | 0.043*** |
|  | (11.15) | (10.24) | (4.04) |
| roa | -0.557*** | -0.740*** | -0.930*** |
|  | (-16.12) | (-28.94) | (-28.18) |
| cashflow | -0.126*** | -0.127*** | -0.128*** |
|  | (-5.13) | (-7.02) | (-5.46) |
| growth | 0.000*** | 0.000*** | 0.000*** |
|  | (12.70) | (12.09) | (5.25) |
| tobinq | 0.001 | 0.003** | 0.005** |
|  | (0.53) | (1.98) | (2.55) |
| top1 | -0.006 | -0.004 | -0.002 |
|  | (-0.63) | (-0.54) | (-0.18) |
| size | 0.078*** | 0.069*** | 0.061*** |
|  | (44.34) | (53.63) | (36.36) |
| listage | 0.023*** | 0.030*** | 0.037*** |
|  | (10.69) | (18.76) | (17.91) |
| m2 | 0.007*** | 0.007*** | 0.007*** |
|  | (10.48) | (14.07) | (10.76) |
| gdp | 0.003*** | 0.003*** | 0.003*** |
|  | (3.30) | (4.28) | (3.15) |
| 时间效应 | 控制 | 控制 | 控制 |
| 行业效应 | 控制 | 控制 | 控制 |
| N | 27737 | 27737 | 27737 |

1.3 异质性

|  | (1) | (2) | (3) | (4) |
| --- | --- | --- | --- | --- |
|  | lev | lev | lev | lev |
| dfi | -0.135*** | -0.137*** | -0.137*** | -0.137*** |
|  | (-13.493) | (-13.690) | (-13.660) | (-13.659) |
| soe×dfi | 0.003*** |  |  |  |
|  | (6.951) |  |  |  |
| sca×dfi |  | 0.001*** |  |  |
|  |  | (2.700) |  |  |
| pro×dfi |  |  | 0.002 |  |
|  |  |  | (1.025) |  |
| hl×dfi |  |  |  | 0.001** |
|  |  |  |  | (2.540) |
| fix | 0.080*** | 0.084*** | 0.084*** | 0.084*** |
|  | (9.445) | (9.907) | (9.943) | (9.894) |
| roa | -0.744*** | -0.744*** | -0.744*** | -0.745*** |
|  | (-27.393) | (-27.306) | (-27.310) | (-27.328) |
| cashflow | -0.124*** | -0.127*** | -0.127*** | -0.127*** |
|  | (-7.169) | (-7.318) | (-7.336) | (-7.331) |
| growth | 0.000*** | 0.000*** | 0.000*** | 0.000*** |
|  | (13.306) | (13.876) | (14.244) | (13.914) |
| tobinq | 0.003*** | 0.003*** | 0.003*** | 0.003*** |
|  | (2.916) | (2.880) | (2.890) | (2.894) |
| top1 | -0.014** | -0.004 | -0.004 | -0.004 |
|  | (-2.156) | (-0.663) | (-0.593) | (-0.647) |
| size | 0.068*** | 0.069*** | 0.069*** | 0.069*** |
|  | (62.011) | (62.930) | (63.151) | (62.985) |
| listage | 0.027*** | 0.030*** | 0.030*** | 0.030*** |
|  | (16.162) | (19.238) | (19.400) | (19.147) |
| m2 | 0.007*** | 0.007*** | 0.007*** | 0.007*** |
|  | (9.945) | (10.492) | (10.555) | (10.492) |
| gdp | 0.003*** | 0.003*** | 0.003*** | 0.003*** |
|  | (3.442) | (3.592) | (3.582) | (3.560) |
| Cons | -0.462*** | -0.477*** | -0.479*** | -0.478*** |
|  | (-7.155) | (-7.387) | (-7.423) | (-7.405) |
| 时间效应 | 控制 | 控制 | 控制 | 控制 |
| 行业效应 | 控制 | 控制 | 控制 | 控制 |
| N | 27739 | 27739 | 27739 | 27739 |
| r2_a | 0.4630 | 0.4621 | 0.4620 | 0.4621 |

注：pro是高于平均growth为1，否则为0。其他的赋值方法与原文一样

1.4 中介效应

Regression Table

|  | (1) | (2) | (3) | (4) |
| --- | --- | --- | --- | --- |
|  | cost2 | KZ指数 | Idiosyncratic_Risk | lev |
| dfi | -0.011** | -1.173*** | -0.002* | -0.076*** |
|  | (-2.293) | (-11.171) | (-1.648) | (-9.196) |
| cost2 |  |  |  | 0.014*** |
|  |  |  |  | (2.611) |
| KZ指数 |  |  |  | 0.055*** |
|  |  |  |  | (79.909) |
| Idiosyncratic_Risk |  |  |  | 0.572*** |
|  |  |  |  | (10.873) |
| fix | 0.009* | 0.571*** | -0.005*** | 0.041*** |
|  | (1.792) | (7.309) | (-6.605) | (6.319) |
| growth | -0.000*** | 0.000*** | 0.000 | 0.000*** |
|  | (-3.768) | (5.384) | (0.660) | (17.300) |
| size | 0.002*** | 0.116*** | -0.003*** | 0.064*** |
|  | (2.715) | (4.955) | (-35.947) | (64.327) |
| listage | 0.006*** | 0.569*** | -0.003*** | 0.001 |
|  | (5.787) | (24.485) | (-17.778) | (0.405) |
| roa | 0.007 | -12.818*** | -0.012*** | -0.085*** |
|  | (1.306) | (-30.729) | (-8.759) | (-3.966) |
| m2 | 0.001** | 0.077*** | 0.001*** | 0.003*** |
|  | (2.379) | (11.117) | (16.244) | (4.698) |
| tobinq | -0.000 | 0.111*** | 0.000*** | -0.003** |
|  | (-0.244) | (3.122) | (11.436) | (-2.254) |
| gdp | 0.000 | 0.036*** | -0.002*** | 0.002*** |
|  | (0.639) | (4.303) | (-23.081) | (3.485) |
| Cons | 0.019 | 3.552*** | 0.145*** | -0.725*** |
|  | (0.602) | (4.556) | (21.403) | (-13.568) |
| 时间效应 | 控制 | 控制 | 控制 | 控制 |
| 行业效应 | 控制 | 控制 | 控制 | 控制 |
| N | 27737 | 27737 | 27257 | 27257 |
| r2_a | 0.0086 | 0.4571 | 0.3195 | 0.6470 |

Cost2=财务费用/营业总成本，反映企业融资成本，来自csmar

Idiosyncratic_Risk反映风险承担能力，从CSMAR获取股票的周收益率和市场的周收益率，回归获取残差，并计算标准差，来反映其风险承担能力。

Kz指数来自csmar数据库，反映企业融资约束水平

各自的中介效应bootstrap检验

|  | 路径 | 系数 | 标准误 | Bootstrap95%置信区间 | |
| --- | --- | --- | --- | --- | --- |
|  |  |  |  | 下限 | 上限 |
| cost2 | 间接效应 | -.0001**^*^** | .0001 | -.0006 | -0.0000 |
|  | 直接效应 | -.1412**^***^** | .0081 | -.1538 | -.1162 |
| KZ指数 | 间接效应 | -.0647**^***^** | .0047 | -.0712 | -.0565 |
|  | 直接效应 | -.0766**^***^** | .0076 | -.0844 | -.0593 |
| Idiosyncratic_Risk | 间接效应 | -.0020**^**^** | .0011 | -.0039 | -.0000 |
|  | 直接效应 | -.1373**^***^** | .0080 | -.1535 | -.1177 |

注：上表所列的置信区间为偏差校正置信区间

1.5 进一步分析

|  | 低市场化程度 | 高市场化程度 | 松监管 | 严监管 |
| --- | --- | --- | --- | --- |
|  | (1) | (2) | (3) | (4) |
|  | lev | lev | lev | lev |
| dfi | -0.149*** | -0.144*** | -0.147*** | -0.136*** |
|  | (-9.033) | (-8.150) | (-9.356) | (-10.686) |
| fix | 0.072*** | 0.078*** | 0.052*** | 0.080*** |
|  | (5.119) | (5.465) | (3.901) | (7.351) |
| roa | -0.766*** | -0.722*** | -1.110*** | -0.669*** |
|  | (-18.738) | (-13.290) | (-25.872) | (-22.078) |
| cashflow | -0.125*** | -0.103*** | -0.073*** | -0.150*** |
|  | (-4.185) | (-3.651) | (-3.036) | (-6.478) |
| growth | 0.000 | 0.000 | 0.000*** | 0.000 |
|  | (0.582) | (0.588) | (11.761) | (0.859) |
| tobinq | 0.002 | 0.006*** | 0.002 | 0.004*** |
|  | (1.477) | (6.689) | (1.164) | (5.993) |
| top1 | 0.030*** | -0.001 | 0.008 | -0.008 |
|  | (2.766) | (-0.052) | (0.794) | (-0.999) |
| size | 0.066*** | 0.075*** | 0.071*** | 0.070*** |
|  | (41.022) | (48.335) | (43.144) | (61.713) |
| listage | 0.035*** | 0.028*** | 0.069*** | 0.008*** |
|  | (14.110) | (10.387) | (28.102) | (4.079) |
| m2 | 0.008*** | 0.005*** | 0.002 | 0.001 |
|  | (7.244) | (4.601) | (0.564) | (1.221) |
| gdp | 0.002 | 0.004*** | 0.017** | 0.001 |
|  | (1.325) | (3.027) | (2.389) | (1.560) |
| Cons | -0.426*** | -0.564*** | -0.542*** | -0.411*** |
|  | (-3.747) | (-5.037) | (-5.353) | (-5.091) |
| 时间效应 | 控制 | 控制 | 控制 | 控制 |
| 行业效应 | 控制 | 控制 | 控制 | 控制 |
| N | 9598 | 9313 | 9989 | 17748 |
| r2_a | 0.4827 | 0.4759 | 0.5517 | 0.4344 |

市场化程度分类标准：若小于市场化指数中位数水平7.35，则为低市场化程度，否则为高市场化程度（来自wind）

监管分类：参考王小燕等( 2019) 在设计政府数字金融监管的相关研究，将 2015 年“互联网金融监管元年”前后设置“0—1”虚拟变量。
