# Supplementary material for: The impact of digital financial development on corporate leverage ratio: The case of a-share listed non-financial enterprises in China’s Shanghai and Shenzhen stock exchanges
Source: PLoS One. 2024 Aug 12;19(8):e0302978. doi: 10.1371/journal.pone.0302978 (PMC11318853; doi:10.1371/journal.pone.0302978)
Supplement: S1 Appendix — (DOCX) [file pone.0302978.s002.docx]

**Appendix 1**

**Variable concept and definitions**

| **Variable** | **Abbreviation** | **Theoretical definition** | **Operational definition** |
| --- | --- | --- | --- |
| Corporate leverage | LEV | Measures total corporate leverage | Total Liabilities ÷ Total Assets |
| Short-term leverage | SLEV | Measures the short-term leverage of an enterprise | Current Liabilities ÷ Total Assets |
| Long-term leverage | LLEV | Measures the long-term leverage of an enterprise | Non-current liabilities ÷ Total assets |
| Digital finance development | DIF | Measures the level of digital finance development | The natural logarithm of the digital financial inclusion index |
| Fixed assets proportion | FIX | Measures the fixed assets of an enterprise | Fixed assets ÷ total assets |
| Return on Assets | ROA | Measures an enterprise's asset utilization | Net profit ÷ Total assets |
| Operating cash flow | Cashflow | Measure the liquidity level of an enterprise | Operating cash flow ÷ total assets |
| Revenue growth rate | GRO | Measures the growth capacity of an enterprise | (Current operating income - last period operating income) ÷ last period operating income |
| Tobin's Q | Tobinq | Measure future investment opportunities of a business | Market Cap ÷ Total Assets |
| Equity concentration | Top1 | Measure the governance structure of an enterprise | The sum of the shareholding ratios of the top ten shareholders |
| Enterprise size | Size | Measure the size of a firm | Natural logarithm of total assets |
| Time to market | Age | Measure the longevity of a business | The natural logarithm of (current year - listing year + 1) |
| Broad money growth rate | M2 | Measure changes in monetary policy | Money growth rate |
| Economic growth rate | GDP | Measure changes in the macroeconomic environment | economic growth rate |
| Industry fixed effect | Industry | Control industry-level differences | Industry dummy variables are set according to the *CSRC's 2012 industry classification standards. Those belonging to the industry take the value of 1, otherwise the value is 0. |
| Time fixed effect | Year | Control the effects of unobservable time factors | Time dummies are set as 1 for year, otherwise 0. |
| *CSRC = China Securities Regulatory Commission | | | |
