# Supplementary material for: The impact of digital financial development on corporate leverage ratio: The case of a-share listed non-financial enterprises in China’s Shanghai and Shenzhen stock exchanges
Source: PLoS One. 2024 Aug 12;19(8):e0302978. doi: 10.1371/journal.pone.0302978 (PMC11318853; doi:10.1371/journal.pone.0302978)
Supplement: Attachment — Submitted filename: Manuscript Number-PONE-D-23-44191.docx [file pone.0302978.s003.docx]

Manuscript Number: PONE-D-23-44191

1、Reading the abstract and introduction section, readers may find it hard to understand the study aims and contributions of the manuscript. The interesting points are vague. The current abstract and introduction section cannot attract readers' interests. The authors need to explain what is interesting and important.

2、Abbreviations that appear for the first time need to be elaborated.

3、Literature review：the main ideas of the literature should be pointed out. The citation presentation and citation format of the literature review need to be changed，please read some related papers for the writing of literature review.

4、H1 is not even a complete sentence.

 5、Some opinions showed up in the paper should add relevant literature to support the point of view to make the further discussions be more convinced for the readers for example: Page 15 “The development of digital finance can not only reduce the degree of financing discrimination suffered by small-scale enterprises, increase their sources of financing, but also stabilize the intermediary income of large-scale enterprises, thereby reducing the leverage ratio of the two. However, compared with small-scale enterprises with relatively low leverage levels, the development of digital finance may significantly impact the leverage ratio of large-scale enterprises with higher leverage levels.”

6、The basis for the selection of control variables and the corresponding references are not mentioned.

7、The data collected only till 2020,would it be possible to retain updated data?

8、A mistake shows up in page 20“The control variables in **column (2)-(2)** show that …..”

9、Table 3, the directions of the coefficients of some control variables are positive or negative in different column, please indicate the specified reason of each variable.

10、The result showed in table 4 is one stage or second stage result? The reason to choose that instrumental variables should be indicated in the main text.

11、The potential reason of the heterogeneity should be explained in detail.

12、Mechanism: Insufficient elaboration of theoretical analyses for mechanism analysis

13、conclusion and recommendation need modification and the authors are suggested to advise such type of suggestion that is practical attainable.

14、Indicate direction for future research and propose some improvements regarding digital financial development on corporate leverage ratio.”

15、Language: It is noted that your manuscript needs careful polishing by someone with expertise in technical English editing so that the goals and results of the study are clear to the reader.

16、Font size and line spacing need to be consistent，Each part heading is too long
