# Supplementary material for: The impact of digital financial development on corporate leverage ratio: The case of a-share listed non-financial enterprises in China’s Shanghai and Shenzhen stock exchanges
Source: PLoS One. 2024 Aug 12;19(8):e0302978. doi: 10.1371/journal.pone.0302978 (PMC11318853; doi:10.1371/journal.pone.0302978)
Supplement: Attachment — Submitted filename: Response to reviewers.docx [file pone.0302978.s004.docx]

**Reviewer 1:**

We are thankful to the respectable Reviewer for sparing his precious time to suggest the necessary action/s required for the improvement of our manuscript.

**Comment:** 1. A literature review of relevant research methodologies is missing from the research synthesis, and it is recommended that it be supplemented.

**Response:** We sincerely appreciate the esteemed reviewer for providing valuable suggestions. In response to the feedback, we have addressed the missing aspect of relevant research methodology in the research synthesis by supplementing it accordingly.

[Please go through the methodology section]

**Comment:** 2. In the manuscript, "H2: The negative impact of digital finance development on corporate leverage ratios is different among state-owned and non-state-owned enterprises." should read H3.

**Response:** We express our gratitude to the esteemed reviewer for their insightful feedback. Following their suggestions. We have rectified this mistake and ensured that Hypothesis 3 is appropriately positioned within the hypothesis development section.

H3: The development of digital finance can reduce the corporate leverage ratios, of both state-owned and non-state-owned enterprises.

[Please go through the hypothesis development section page #13]

**Comment:** 3. This study focuses on the heterogeneity of the impact of the development of digital finance on the leverage ratios of state-owned and non-State-owned enterprises, but the "Policy recommendations" section does not explicitly propose differentiated regulatory strategies for different types of enterprises.

**Response:** We express our gratitude to the esteemed reviewer for their insightful feedback. In response, we have added the policy recommendation for both state-owned and non-state-owned enterprises as follows:.

The digital finance development on corporate leverage ratios across different types of enterprises. By implementing the suggested recommendations, State-owned enterprises in China often have access to preferential lending rates and support from the government, which can result in higher leverage ratios compared to non-state-owned enterprises. On the other hand, non-state-owned enterprises may face more stringent lending conditions and higher borrowing costs, leading to lower leverage ratios. This study further policy recommended to Implement a standardized and transparent framework for determining corporate leverage ratios, applicable to both state-owned and non-state-owned enterprises. This framework should consider the specific characteristics and risks of different industries, while also ensuring consistency in measuring leverage ratios across enterprises.

**Comment:** 4. Please identify the software platform used for the calculation of the econometric model and provide further details on the rationale for the econometric model.

**Response:** We express our gratitude to the esteemed reviewer for their insightful feedback. based on hypotheses development, in this study, we employ an econometric approach utilizing panel data regression analysis to examine the relationship between digital finance development and leverage ratios. Stata software is chosen as the analytical tool for its robust capabilities in handling panel data and estimating regression models. The rationale for selecting this econometric model lies in its ability to control for unobserved heterogeneity across entities and time periods, providing a more accurate estimation of the relationship between digital finance development and leverage ratios. By including control variables indicating enterprises characteristics, industry effects, and time trends, we aim to capture the complex nature of the relationship and generate insights into how digital finance impacts leverage ratios among various enterprises.

[Please go through the methodology section Pag #7]

**Comment:** 5. It is recommended that a technology roadmap be added to visualize the framework of the paper.

**Response:** We express our gratitude to the esteemed reviewer for their insightful feedback. Following their suggestions, we have incorporated both technology roadmap and framework of the paper as well.

The remainder of the study is organized as follows: Section 2 literature review and research Hypotheses Section 3 discusses the theoretical framework and, empirical methodology. Section 4 analyzes the empirical results. Section 5 conducts the mechanism analysis and heterogeneity analysis. Section 6 External economic impact of digital finance and leverage ratio Section 7 conclusions and policy recommendations.

**Fig 1. Conceptual model**

Note: Hypothesis: H1: H1: The development of digital finance can reduce corporate leverage ratio of enterprises. Hypothesis: H2: The development of digital finance can reduce corporate leverage ratios, affecting both large-scale and small-scale enterprises, and high-leverage and low-leverage enterprises. Hypothesis: H3: The development of digital finance can reduce the corporate leverage ratios, of both state-owned and non-state-owned enterprises. Hypothesis: H4: digital finance reduces the enterprise's financial risk by alleviating the financing constraint.

**Comment:** 6. Please explain the need for the study "6. The impact of the external economic environment on the relationship between digital finance development and corporate leverage ratio" in the manuscript and clarify the role of the research in the sixth part of the article in the overall context.

### **Response:** We express our gratitude to the esteemed reviewer for their insightful feedback. In response, we have added The impact of the external economic environment on the relationship between digital finance development and corporate leverage ratio" as follows:.

### **External economic impact of digital finance and leverage ratio**

The study on the impact of the external economic environment on the relationship between digital finance development and corporate leverage ratio is crucial for understanding the potential dynamic between digital finance, the external economic context, and corporate financial structures. Digital finance, by offering alternative fund of sources and improving financial efficiency, has the potential to reduce corporate leverage ratios [1] However, the extent to which this occurs can be change by the external economic environment [2] In addition, the degree of financial supervision can influence the risk management decision of enterprises and the overall solvency of the financial mechanism. By focusing on how factors such as the degree of marketization and financial supervision influence the relationship between digital finance and corporate leverage ratios, the study conducted based on previous model by[3, 4]. This study further provides valuable insights into the mechanisms through which digital finance may mitigate leverage. Through rigorous analysis, this study not only explains the potential benefits of digital finance in reducing corporate leverage but also emphasizes the importance of regulatory environments and market conditions in shaping the effectiveness of digital financial solutions.

**Reviewer 2:**

We are thankful to the respectable Reviewer for sparing his precious time to suggest the necessary action/s required for the improvement of our manuscript.

**Comment:** 1、Reading the abstract and introduction section, readers may find it hard to understand the study aims and contributions of the manuscript. The interesting points are vague. The current abstract and introduction section cannot attract readers' interests. The authors need to explain what is interesting and important.

**Response:** Thanks for the comment. We have further refined the abstract and introduction section to reinforce the paper’s quality. as follows

**Abstract**

This study investigates the impact of digital finance on corporate leverage ratios. The study employed a large sample of China's Shanghai and Shenzhen A-share non-financial listed enterprises from 2011-2020. The study's results depict that the development of digital finance can significantly reduce the leverage ratio of enterprises. We empirically identified that digital finance affects the difference in the term structure of the corporate leverage ratio. It was found that the development of digital finance has a significant negative impact on enterprises' short-term and long-term leverage ratios. Moreover, our heterogeneity analysis shows that the negative effect of digital financial development on corporate leverage ratios is different in state-owned and non-state-owned enterprises, large-scale and small-scale enterprises, and high-leverage and low-leverage enterprises. Mechanism analysis shows that the development of digital finance can reduce corporate leverage by lowering financing costs, alleviating financing constraints, and weakening non-systemic risks. Therefore, policymakers should focus on developing and adopting digital finance by creating a supportive regulatory environment, improving access to digital financial services, and encouraging innovation in the digital finance sector. Finally, our results remain robust after addressing endogeneity issues and conducting robustness checks.

The study aims and contributions of the manuscript

Firstly, it examines the influence of digital finance development on corporate leverage ratios at the micro-enterprise level. It elevates research on digital finance and how to impact insights for effectively reducing enterprise leverage ratios.

Second, we attempted to contribute the latest literature that investigates the impact of digital finance on corporate financial leverage performance; specifically, we employ a non-financial enterprise-level analysis. and the role of DIF in affecting firms' financial activities, and extend previous studies from the perspective of financial innovation and sustainability

Third, this study provides more detailed results on the intermediate mechanisms by which digital finance can digitally manage firms' financial leverage ratios to reduce financial risk and constraints. This study further contributes to the enterprise-level data to test the mechanism between digital finance and corporate leverage, fulfilling the lack of experimental evidence.

Fourth, the mechanism of digital finance development on corporate leverage ratios should provide solutions for preventing and mitigating a series of risks that excessive corporate leverage ratios may cause.

Finally, it tests the impact of the external economic environment on the relationship between digital finance development and corporate leverage ratio, which can provide a basis for what measures the government takes to effectively play the positive role of digital finance.

[Please go through the abstract and Introduction section Page 1-3]

**Comment:** 2、Abbreviations that appear for the first time need to be elaborated

**Response:** We express our gratitude to the esteemed reviewer for their insightful feedback. In response, we have elaborated abbreviations that appear for the first time,

[Please go through the entire of the paper]

**Comment:** 3、Literature review：the main ideas of the literature should be pointed out. The citation presentation and citation format of the literature review need to be changed please read some related papers for the writing of literature review.

**Response:** We express our gratitude to the esteemed reviewer for their insightful feedback. In response, we have updated the literature review as follows.

The The research on the development of digital finance on corporate leverage ratio has been deepening since Fisher [31] constructed the debt-deflation theory, which has become the focus of discussions in the field of macroeconomics and corporate finance at the macro level. Furthermore, many scholars conducted in their prior studies on the economic effects of digital finance specifically focused on macro and micro-economic perspectives [13, 14]. In addition, some studies empirically analyzed the impact of poverty reduction, such as Li et al [19], and Das and Chatterjee [32]. Digital finance inclusive and digital growth [33]. For example, some researchers have empirically analyzed digital finance in the corporate banking sector [34], corporate innovation [35], financial risk [23], financial stability [36], and financial innovation [22]. From the perspective of business evolution, digital finance development has enhanced competition among the financial sectors [37]. However, digital technology in the financial industry can bring financial risks [23].

From a business perspective, scholars also incorporated the economic benefits of digital finance on financial development into their studies, analysed the correlation between digital finance and financial inclusion, and identified economic indicators such as digital finance relations and financial stability [38], financial efficiency [39], corporate financial fraud [4], and financial distress [21]. Furthermore, some research scholars significantly clarify the benefits of digital finance in the context of social and environmental perspectives, such as corporate ESG [20, 40] analyzed the benefits of digital finance on carbon emissions and clarified that the impact of digital finance can significantly reduce carbon emissions in specific sectors. However, few studies have been analyzed on microeconomic indicators [41]. Few researches have been conducted on household consumption [18], and few studies have been examined from the perspective of digital finance on corporate innovation [41, 42]. In addition, few scholars address the impact of digital finance from the perspective of corporate financial constraint, efficiency, and performance [28, 43]; beyond rare studies have focused on digital finance on the financial efficiency of firms.

On the other hand, most of the latest studies empirically conducted by different scholars on digital financial development from the perspective of enterprises' financial control, such as Sun et al [4], corporate financial distress [21], enterprises financial deleverage [44], but few research studies have explored the impact factor of corporate leverage [45]. Scholars have clarified that corporate leverage investment can significantly improve corporate value [45]. Operating performance can reduce financing costs, enhance corporate investment performance and promote financial innovation to mitigate financial risk [46], after verifying the important of corporate leverage ratios investment and further including what factors impact corporate leverage performance and how to enhance corporate operating efficiency and achieve financial development by improving corporate leverage ratios performance. This study explores the performance of digital finance and corporate leverage ratios to answer these questions before conducting the empirical analysis. Our proposed research hypotheses are given below. However, limited attention has been paid to identifying the determinants of corporate leverage performance and strategies for enhancing corporate operating efficiency to achieve financial development through improved leverage ratios. Thus, this study aims to fill this gap by examining the relationship between digital finance performance and corporate leverage ratios, thereby shedding light on key drivers of corporate financial health and efficiency in the digital era. This revision aims to provide a more structured and coherent overview of the literature, emphasizing the gap in existing research and the specific focus of the current study. Additionally, it suggests directions for future research by highlighting the importance of understanding the determinants of corporate leverage performance and strategies for enhancing corporate operating efficiency in the context of digital finance.

**Comment:** 4、H1 is not even a complete sentence.

**Response:** We express our gratitude to the esteemed reviewer for their insightful feedback. Following their suggestions. We have rectified this mistake and ensured that Hypothesis 1 is appropriately positioned within the hypothesis development section.

**H1** The development of digital finance can reduce corporate leverage ratio of enterprises.

**Comment:** 5、Some opinions showed up in the paper should add relevant literature to support the point of view to make the further discussions be more convinced for the readers for example: Page 15 ”The development of digital finance can not only reduce the degree of financing discrimination suffered by small-scale enterprises, increase their sources of financing, but also stabilize the intermediary income of large-scale enterprises, thereby reducing the leverage ratio of the two. However, compared with small-scale enterprises with relatively low leverage levels, the development of digital finance may significantly impact the leverage ratio of large-scale enterprises with higher leverage levels.” )

**Response:** We express our gratitude to the esteemed reviewer for their insightful feedback. In response, We have rectified this mistake. As follows below.

different levels of financing discrimination incentivize large-scale firms with lower loan costs to engage in financial markets as financial intermediaries for intermediary benefits, which not only expands the scale of shadow banking but also makes large-scale enterprises more leveraged [5, 6] The development of digital finance can not only reduce the degree of financing discrimination faced by small-scale enterprises, but also stabilize the intermediary income of large-scale enterprises, However, under the influence of these two aspects, the development of digital finance may have a greater impact on the leverage ratio of large-scale enterprises with higher leverage levels than small and medium scale enterprises with relatively low leverage levels. This results in digital finance development having a relatively sizeable deleveraging effect on high-leverage enterprises. Based on the above analysis, the following research hypothesis is proposed.

**Comment:** 6、The basis for the selection of control variables and the corresponding references are not mentioned.

**Response**. We express our gratitude to the esteemed reviewer for their insightful feedback. In response, We have inserted corresponding references for control variables. As follows below.

Control variables [20, 26, 75]

**Comment:** 7、The data collected only till 2020, would it be possible to retain updated data?
**Response:** Thank for your question but, The development of digital finance with recently updated data from Peking University, encompassing the period up to 2020.

**Comment:** 8、A mistake shows up in page 20“The control variables in column (2)-(2) show that…..”
**Response:** Thanks for the comment. We have incorporated the mistake.

**Comment:** 9、Table 3, the directions of the coefficients of some control variables are positive or negative in different column, please indicate the specified reason of each variable.

**Response:** . We express our gratitude to the esteemed reviewer for their insightful feedback. In response we have indicated the specified reason of each variable as follows.

The descriptive statistics presented in Table 2 highlight substantial variability in the leverage ratios among the sampled firms, with a mean leverage of 0.430 and a notable difference in preference for short-term debt (mean short-term leverage ratio of 0.34) as compared to long-term debt (mean long-term leverage ratio of 0.09). This disparity underscores the strategic financial decisions that enterprises are making in the context of digital financial development, which itself averages a relatively high and narrow value of 5.91. When examining control variables, there is a right-skewed distribution in fixed assets, operating income growth, Tobin's Q, and economic growth, indicating that a minority of firms possess exceptionally high values in these areas. In contrast, variables such as listing time and currency growth rate display a left-skewed distribution. The congruence between average and median values for return on assets, operating cash flow, equity concentration, and enterprise size suggests these variables are normally distributed within the sample. This overview not only reveals the intricate implications of digital finance in shaping corporate financial leverage but also highlights the need for firms to leverage flexible financial strategies that can accommodate the dynamic interplay of technological advancement and economic indicators.

**Comment:** 10、The result showed in table 4 is one stage or second stage result? The reason to choose that instrumental variables should be indicated in the main text.

**Response:** Thank you for your comments we have incorporated the comments

**Comment:** 11、The potential reason of the heterogeneity should be explained in detail.

**Response:** Thank you for your comments we have incorporated the potensial reason heterogeneity. as follows

Heterogeneity Since China is a vast country with huge differences in natural geographic conditions and economic development levels in different regions, the influence of digital financial development on corporate leverage ratios to achievement of distinct enterprises may vary greatly. Through heterogeneity analysis, this study can inspect the causality between corporate leverage ratios improvement and digital financial inclusion more thoroughly, to better promote sustainable corporate financial development.

**Comment:** 12、Mechanism: Insufficient elaboration of theoretical analyses for mechanism analysis

**Response:** Thanks for the comment. We have further improved the theoretical analyses for mechanism analysis as follows:

3.2.3 Mechanism analysis Based on hypotheses development, in this study, we employ an econometric approach utilizing panel data regression analysis to examine the relationship between digital finance development and leverage ratios. Stata software is used as the analytical tool for its robust capabilities in handling panel data and estimating regression models. The rationale for selecting this econometric model lies in its ability to control for unobserved heterogeneity across entities and time periods, providing a more accurate estimation of the relationship between digital finance development and leverage ratios. By including control variables indicating enterprises characteristics, industry effects, and time trends, we aim to investigate the multifaceted nature of the relationship and generate insights into how digital finance impacts leverage ratios among various enterprises. Drawing on the research methods of existing literature [7], the model is specified as follows. Financing constraint: by re- Ferring to Hadlock and Pierce [8], this paper employs the SA index to measure the financing constraints of enterprises. The specific calculation formula is as follows Where the mediation variables are financing cost (cost), financing constraint (K.Z.) and non-systemic risk (risk), which are determined by the following formulas, cost = financial expenses divided by total operating income; K.Z. comes from CSMAR database; risk reflects non-systemic risk, which is obtained by from the weekly yield of the stock and the weekly return of the market from CSMAR to regress the residuals and calculate the standard deviation to reflect the unsystematic risk profile.

**Comment:** 13、conclusion and recommendation need modification and the authors are suggested to advise such type of suggestion that is practical attainable.

**Response:** Thanks for the comment. We have further improved and changed the conclusion and recommendation as follows:

## **7.1 Conclusions**

Based on a sample of 2011-2020 A-share non-financial listed companies in China, this paper examines the impact and mechanism of digital finance development on corporate leverage. The following conclusions can be drawn from the research findings: Firstly, digital finance development shows a significant negative correlation with the corporate leverage ratio. Moreover, after considering the difference in the term structure of corporate leverage ratio, it found that digital finance development exhibits a significant negative correlation with both short-term and long-term corporate leverage ratios. This suggests that digital finance development contributes to reducing the leverage ratio of enterprises, be it short-term or long-term leverage. Secondly, the negative impact of digital financial development on corporate leverage is heterogeneous due to different characteristics of enterprises, including state-owned and non-state-owned enterprises, large and small-scale enterprises, and high-leverage and low-leverage enterprises. Third, Mechanism analysis demonstrates that the development of digital finance can reduce the corporate leverage ratio by reducing financing costs, easing financing constraints, and weakening non-systemic risks. Finally, Research on the external economic environment suggests that actively promoting the process of marketization and appropriately strengthening financial supervision will help enhance the positive impact of digital financial development on enterprise deleveraging.

Policy recommendation

Based on the above analysis, this paper puts forward the following policy suggestions: First, increase policy support and actively promote the marketization process to improve the deleveraging effect of digital finance. The development of digital finance can not only reduce the leverage ratio of enterprises, but also the effect of deleveraging is affected by the degree of marketization. Therefore, increasing policy support for the development of digital finance, establishing, and improving the infrastructure for the development of digital finance, and actively promoting the process of marketization will help to better play the deleveraging role of digital finance. Second, innovate digital financial supervision and guide the healthy development of digital finance, to strengthen the positive effect of digital finance on corporate deleveraging. The development time of digital finance is short, and its development model is not yet mature, and financial supervision needs to be properly strengthened to regulate and guide its healthy development. Therefore, to effectively play the role of the development of digital finance in deleveraging enterprises, the regulatory authorities should innovate the financial supervision model while strengthening the supervision of digital finance and preventing its potential risks, to ensure the healthy development of digital finance and strengthen its positive effect. Third, State-owned enterprises in China often have access to preferential lending rates and support from the government, which can result in higher leverage ratios compared to non-state-owned enterprises. On the other hand, non-state-owned enterprises may face more stringent lending conditions and higher borrowing costs, leading to lower leverage ratios. Finally, the development of digital finance as an opportunity to promote digital links between financial institutions and enterprises, so that enterprises can actively participate in deleveraging. Information has become a key element in the development of modern enterprises. By strengthening the digital connection between financial institutions and enterprises, on the one hand, financial institutions can timely and effectively understand the operating conditions of enterprises, on the other hand, it can improve the information transparency among economic agents, and enable enterprises to actively participate in deleveraging and control the leverage ratio within a reasonable range.

**Comment:** 14、Indicate direction for future research and propose some improvements regarding digital financial development on corporate leverage ratio.”

**Response:** Thank you for your comments we have incorporated the future research and propose some improvements regarding digital financial development on corporate leverage ratio. as follows

This study empirically analyses the relationship between the development of digital finance and corporate leverage ratios within the existing theoretical framework and data availability. However, due to the initial stage of corporate leverage ratio’s disclosure and the lack of leverage data by many listed firms, the available data for this study has some limitations. In addition, we examine the mechanism through which digital finance affects corporate leverage ratios. There may be another possible technique through which digital finance may have an impact on corporate leverage performance. Future studies can be conducted from the perspectives of corporate financing performance, internal audit mechanisms, and digital finance. corporate liquidity management Finally, while this study focuses on the improvement that digital finance brings to enterprises leverage ratio performance, it would also be worthwhile to evaluate the positive impact of digital finance on firms’ operational performance. All these highlighted problems above are worth to recommending in depth for future research.

**Comment:** 15、Language: It is noted that your manuscript needs careful polishing by someone with expertise in technical English editing so that the goals and results of the study are clear to the reader.

**Response:** Correction incorporated

**Comment:** 16、Font size and line spacing need to be consistent Each part heading is too long

**Response:** Correction incorporated

**Reviewer 3:**

We are thankful to the respectable Reviewer for sparing his precious time to suggest the necessary action/s required for the improvement of our manuscript.

**Comment:** - The motivation and reasoning for the study are clearly stated in the introduction. However, the literature review could develop the motivation of the study in greater detail. Thus, the need to estimate digital finance's impact on the leverage ratio before hypothesis 1 is introduced could be presented in more detail.

**Response:** We sincerely appreciate the esteemed reviewer for providing valuable suggestions. In response to the feedback, we have incorporated the litureture review in a detail before hypothesis 1 as follows

The research on development of digital finance on corporate leverage ratio has been deepening since Fisher [9] constructed the debt-deflation theory has become the focus of discussions in the field of macroeconomics and corporate finance at the macro level, and many scholars study the economic effects of digital finance specifically focused on macro and micro-economic perspectives, such as the empirical studies conducted by [10, 11]. In addition, some studies empirically analysed the impact of poverty reduction, such as [12, 13]. Digital finance is inclusive and digital growth [14]. For example, some researchers have empirically analysed digital finance in the corporate banking sector [15]. Corporate innovation [16]. Financial risk [17]. financial stability [18], and financial innovation [19]. From the perspective of business evolution, digital finance development has enhanced competition among the financial sectors [20]. However, digital technology in the financial industry can bring financial risks [17]. From a business perspective, scholars also incorporate the economic benefits of digital finance on financial development into their studies, analyse the correlation between digital finance and financial inclusion, and identify economic indicators such as digital finance relations and financial stability. [21], financial efficiency [22], corporate financial fraud [23] financial distress [24]. furthermore some research scholars clarify the benefits of digital finance significantly in the context on social and environmental perspective such as corporate ESG. [25, 26] analysed the benefits of digital finance on carbon emission and clarify that the impact of digital finance can significantly reduce carbon emission in specific sector. However, few studies have been analysed on microeconomic indicators [27]. Few researches have been conducted in the context of household consumption [28], and few studies have been analysed from the perspective of digital finance on corporate innovation [27, 29] in addition few scholars address the impact of digital finance from the perspective of corporate financial constraint, efficiency, and performance [30, 31] beyond rare studies have focused on the digital finance on financial efficiency of the firms. On the other hand, most of latest studies empirically conducted by different scholars on digital financial development from the perspective of enterprises' financial control, such as [23], corporate financial distress [24], enterprises financial deleverage [32] but few research studies have explored the impact factor of corporate leverage [33]. Scholar have clarified that corporate leverage investment can significantly improve corporate value [33], and operating performance can reduce financing costs enhance corporate investment performance and promote financial innovation to reduce financial risk [34] after verifying the important of corporate leverage ratios investment and further include what factor impact corporate leverage performance and how to enhance corporate operating efficiency and achieve financial development by improving corporate leverage ratios performance. This study explores the performance of digital finance and corporate leverage ratios to answer these questions before conducting the empirical analysis. Our proposed hypothesis is given below. However, limited attention has been paid to identifying the determinants of corporate leverage performance and strategies for enhancing corporate operating efficiency to achieve financial development through improved leverage ratios. Thus, this study aims to fill this gap by examining the relationship between digital finance performance and corporate leverage ratios, thereby shedding light on key drivers of corporate financial health and efficiency in the digital era. This revision aims to provide a more structured and coherent overview of the literature, emphasizing the gap in existing research and the specific focus of the current study. Additionally, it suggests directions for future research by highlighting the importance of understanding the determinants of corporate leverage performance and strategies for enhancing corporate operating efficiency in the context of digital finance

**Comment:** - Additionally, Hypothesis 1 must be reformulated. The formulation “There exists the significant impact of digital financial development on corporate leverage ratios” would probably be better.

**Response:** Thank you for your comment we have incorporated the Hypothesis 1 as follows

H1: The development of digital finance can reduce corporate leverage ratio of enterprises.

**Comment:** - The reasoning for hypotheses 2 and 3 should include the particularity of the case of China.

**Response:** Thank you for your comment we have incorporated the reasoning for hypotheses 2 and 3 are particularity of the case of China.

[Please go through the hypothesis development section page #13]

**Comment:** - The reasoning for hypothesis 3 is a little mixed up. If the hypothesis focuses on state vs. non-state enterprises, the argumentation for large vs. small enterprises should not be included in the description. On the other side, based on the presentation of the results, an additional hypothesis regarding firm size could be introduced.

**Response:** Thank you for your comment, we have developed the hapothisis for both state vs. non-state enterprises, and large vs. small enterprises sepratly and additional hypothesis regarding firm size are developed as follows

*2.3.1 Firms' sizes and leverage level*

The trade-off theory indicates that corporate leverage is determined by the costs of bankruptcy and agency problems, necessitating the incurrence of corporate liabilities for the tax shield benefits [35]. For high-leverage enterprises, debt levels may exceed the reasonable threshold, resulting in high costs. Therefore, the expected bankruptcy costs and agency costs caused by their high debt higher than those of low-leverage enterprises. Additionally, because the tax shield income has a marginal decreasing effect, the marginal revenue brought by the increase in the debt of high-leverage enterprises is less than that of low-leverage enterprises. Under the influence of these factors, the development of digital finance may impact the leverage ratio of enterprises by optimizing resource allocation and reducing the costs of enterprise bankruptcy and agency problems. As a result, the deleveraging losses and benefits of high-leverage enterprises are smaller than those of low-leverage enterprises. Small-size enterprises on financing conditions and interest charges has been widely studied in the literature [36]. Some researchers have found that large enterprises, due to their strong market reputations and access to capital, tend to have better financing conditions and lower interest charges. On the other hand, small scale enterprises often face more financial constraints and must contend with unfavourable debt conditions and higher interest charges. Therefore, inventors are more confident in large scale enterprises as their portfolios are more secure, diversify and hence have a lower risk of defaults. Nyeadi and Sare [37] documented that different corporate leverage levels significantly affect enterprises profit. enterprises with high leverage suffer from the higher cost of short-term debt [37, 38]. Such firms mostly use internal sources to repay debt and have to rely on external debt for working capital [39]. However, Lenders have severe liquidity concerns with high-leverage firms because a significant portion of liquid assets is required to pay back debt charges [40]. High-leverage firms already suffer from heavy debt load and lenders are not willing to provide further loans with normal interest rates, so these firms have to pay premium interest rates on further debt. Also, these firms have a high risk of insolvency and bankruptcy, so may have limited investment opportunities. As a result, these firms are not able to earn as much profit as low-leverage firms. High interest charges further reduce profit. In addition different levels of financing discrimination incentivize large-scale firms with lower loan costs to engage in financial markets as financial intermediaries for intermediary benefits, which not only expands the scale of shadow banking but also makes large-scale enterprises more leveraged [5, 6] The development of digital finance can not only reduce the degree of financing discrimination faced by small-scale enterprises, but also stabilize the intermediary income of large-scale enterprises, However, under the influence of these two aspects, the development of digital finance may have a greater impact on the leverage ratio of large-scale enterprises with higher leverage levels than small and medium scale enterprises with relatively low leverage levels. This results in digital finance development having a relatively sizeable deleveraging effect on high-leverage enterprises. Based on the above analysis, the following research hypothesis is proposed.

H2: The development of digital finance can reduce corporate leverage ratios, affecting both large-scale and small-scale enterprises, and high-leverage and low-leverage enterprises.

**Comment:** - For the case of the research design, the reasoning for filtering the data could be explained in greater detail. For example, why specifically did you choose to delete ST firms and financial and real estate segments?

**Response:** Thank you for your comment we have organized the data based on heterogeneity, therefore excluding data of financial companies and real estate firms helps simplify the analysis by focusing on a more specific group. Non-financial companies may have different financial structures and needs compared to those in the financial sector. Similarly, real estate firms operate in a distinct market with unique financial dynamics. By excluding these groups, we can create a more uniform dataset, making it easier to identify patterns and relationships related to digital financial development and corporate leverage. This focused approach improves the clarity and accuracy of our analysis.

**Comment:** - Additionally, the results of the tests of the model assumptions need to be presented in the appendix.

**Response:** Correction incorporated

**Comment:** - It would be helpful if you included the source of each of your variables in Table.

**Response:** Thank you for your comment we have inserted the source of each of variables as follows

| **Role** | **Variable** | **N** | **Mean** | **SD** | **Min** | **Max** |
| --- | --- | --- | --- | --- | --- | --- |
| Dependent variables [41] | LLEV | 27,739 | 0.430 | 0.210 | 0.0100 | 1.760 |
|  | SLEV | 27,739 | 0.340 | 0.180 | 0 | 1.400 |
|  | LLEV | 27,739 | 0.0900 | 0.0800 | -0.0900 | 0.850 |
| Key independent variables [42] | DIF | 27,739 | 5.910 | 0.100 | 5.700 | 6.070 |
| Control variables [25, 41, 43] | FIX | 27,739 | 0.210 | 0.160 | 0 | 0.970 |
|  | ROA | 27,739 | 0.0400 | 0.0800 | -1.860 | 0.880 |
|  | Cashflow | 27,739 | 0.0500 | 0.0800 | -1.940 | 0.880 |
|  | Growth | 27,739 | 5.750 | 813.2 | -1.310 | 130000 |
|  | Tobinq | 27,739 | 2.160 | 2.870 | 0.150 | 259.1 |
|  | Top1 | 27,739 | 0.350 | 0.150 | 0 | 0.900 |
|  | Size | 27,739 | 22.19 | 1.330 | 15.58 | 28.64 |
|  | Listage | 27,739 | 2.150 | 0.800 | 0 | 3.430 |
|  | M2 | 27,739 | 11.40 | 3.350 | 6.990 | 18.95 |
|  | GDP | 27,739 | 6.740 | 2 | 2.350 | 10.60 |

**Comment:** - Probably, already when presenting descriptive results some possible interpretation for specific distributions could be provided (chapter 4.1).

**Response:** Thank you for your comment, we have incorporated descriptive results of possible interpretation for each variables as follows.

The descriptive statistics presented in Table 2 highlight substantial variability in the leverage ratios among the sampled firms, with a mean leverage of 0.430 and a notable difference in preference for short-term debt (mean short-term leverage ratio of 0.34) as compared to long-term debt (mean long-term leverage ratio of 0.09). This disparity underscores the strategic financial decisions that enterprises are making in the context of digital financial development, which itself averages a relatively high and low value of 5.91. When examining control variables, there is a right-skewed distribution in fixed assets, operating income growth, Tobin's Q, and economic growth, indicating that a minority of firms possess exceptionally high values in these areas. In contrast, variables such as listing time and currency growth rate display a left-skewed distribution. The congruence between average and median values for return on assets, operating cash flow, equity concentration, and enterprise size suggests these variables are normally distributed within the sample. This overview not only reveals the intricate implications of digital finance in shaping corporate financial leverage but also highlights the need for firms to leverage flexible financial strategies that can accommodate the dynamic interplay of technological advancement and economic indicators.

**Comment:** - It can be suggested that some of the robustness checks can be moved to the appendix to improve the readability of the paper.

**Response:** Correction incorporated

**Comment:** - It is unclear where some of the results in chapter 4.4 are presented, as I could not see them in Table 8. These relate to the following indicators “…development and the leverage ratio of state-owned enterprises is -0.133, which is also significant at 1% level”, “…while for large-scale enterprises, the regression coefficient is -0.136, which is also significant at a 1% level”. Please, re-check this.

**Response:** Thank you for your comments, we have incorporated the above Correction

**Comment:** - For the case of chapters 5, the reasoning for introducing these analyses is not clear, as it is not reflected in the theoretical part of the paper and the hypotheses. Please, reconsider the necessity of their inclusion in the main part of the paper, the reasoning for their inclusion and probably add the relevant hypotheses/reasoning in the theoretical part. In the current state the theoretical and the analytical parts are not completely aligned.

**Response:** We sincerely appreciate the esteemed reviewer for providing valuable suggestions. In response to the feedback, we have reconsidered relevant hypotheses/reasoning in the theoretical part as follows.

3.2.3 Mechanism variable

Financing constraint: by re- Ferring to Hadlock and Pierce [8], this paper employs the SA index to measure the financing constraints of enterprises. The specific calculation formula is as follows Where the mediation variables are financing cost (cost), financing constraint (K.Z.) and non-systemic risk (risk), which are determined by the following formulas, cost = financial expenses divided by total operating income; K.Z. comes from CSMAR database; risk reflects non-systemic risk, which is obtained by from the weekly yield of the stock and the weekly return of the market from CSMAR to regress the residuals and calculate the standard deviation to reflect the unsystematic risk profile.

**2.4 Mechanism of digital finance effecting corporate leverage ratios**

The main purpose of this study is not only identifying the relationship between the development of digital finance on corporate leverage ratios but also examine the how the digital finance reduces the corporate leverage by lowering financing costs, alleviating financing constraints, and weakening non-systemic risks. The development of Digital finance offers several benefits for businesses in reducing financial risk and alleviating the financing constraint. Firstly, digital finance provides businesses with access to alternative sources of funding [10,63]. These sources include crowdfunding platforms, peer-to-peer lending platforms, and online marketplaces. By diversifying their sources of financing, businesses can reduce their reliance on traditional financial institutions and increase the chances of securing funding for their operations. Second Digital finance plays a crucial role in alleviating financing constraints for enterprises. It provides new financing channels, such as crowdfunding platforms or peer-to-peer lending, which can connect enterprises with potential investors or lenders who are specifically interested in supporting enterprises [44] These platforms use digital technologies to facilitate the matching of capital with enterprises, reducing the barriers to access funding. Additionally, digital finance allows enterprises to reach a larger pool of investors or lenders beyond their local industry [45]. This increased access to funding sources expands the opportunities for enterprises to secure the financing they need to grow and developed. Furthermore, digital finance platforms often offer faster and more streamlined loan application processes compared to traditional financial institutions. These speeds up the funding process and reduces the time and effort required for enterprises to obtain financing. In addition, digital finance tools enable enterprises to automate financial processes, such as invoice management and payment collection, reducing the risk of errors or delays. This automation increases financial efficiency and reduces the likelihood of financial mismanagement or fraud, thereby minimizing financial risk for the enterprise[40]. Finally, Digital finance solutions, such as financial management software or dashes, provide real-time data on various financial metrics, allowing enterprises to monitor and assess their financial health on an ongoing basis. This real-time access to financial data enables timely identification of potential risks and the ability to take proactive measures to mitigate them [46]. This includes identifying cash flow issues, monitoring debt levels, assessing liquidity positions, and evaluating financial ratios [47], Moreover, digital finance reduces the enterprise's financial risk by alleviating the financing constraint, streamlining financial processes, enabling real-time access to critical financial data, and enhancing risk assessment and monitoring capabilities through big data analytics.

H4: digital finance reduces the enterprise’s financial risk by alleviating the financing constraint.

**Comment:** - Limitations of the study as well as the embedding of the results in current theoretical discussion should be added to chapter 7.

**Response:** We sincerely appreciate the esteemed reviewer for providing valuable suggestions. In response to the feedback we have incorporated both Limitations of the study as well as the embedding of the results in current theoretical discussion in the chapter 7 as follows below

This study empirically analyses the relationship between the development of digital finance and corporate leverage ratios within the existing theoretical framework and data availability. However, due to the initial stage of corporate leverage ratio’s disclosure and the lack of leverage data by many listed firms, the available data for this study has some limitations. In addition, we examine the mechanism through which digital finance affects corporate leverage ratios. There may be another possible technique through which digital finance may have an impact on corporate leverage performance. Future studies can be conducted from the perspectives of corporate financing performance, internal audit mechanisms, and digital finance. corporate liquidity management Finally, while this study focuses on the improvement that digital finance brings to enterprises leverage ratio performance, it would also be worthwhile to evaluate the positive impact of digital finance on firms’ operational performance. All these highlighted problems above are worth to recommending in depth for future research.

**Comment:** - Generally, it is necessary to re-read the paper for typos and formatting errors, as some could be identified in the paper (e.g., words “Table 9” at the beginning of two paragraphs on page 26).

**Response:** We sincerely appreciate the esteemed reviewer for providing valuable suggestions. In response to the feedback we have incorporated the paper for typos and formatting errors as well as the “Table 9” at the beginning of two paragraphs on page 26).

References

1.	Guo H, Ye B, Chen Y, Lin W, Guan X, Mao R. Investor attention and corporate leverage manipulation. Finance Research Letters. 2024;60:104914. doi: <https://doi.org/10.1016/j.frl.2023.104914>.

2.	Nikkinen J, Piljak V, Rothovius T. Impact of the 2008–2009 financial crisis on the external and internal linkages of European frontier stock markets. Global Finance Journal. 2020;46:100481. doi: <https://doi.org/10.1016/j.gfj.2019.100481>.

3.	Zhang R, Lin Y, Kuang Y. Will the governance of non-state shareholders inhibit corporate social responsibility performance? Evidence from the mixed-ownership reform of China’s state-owned enterprises. Sustainability. 2022;14(1):527.

4.	Sagner JS. Financial regulation: what should be done? Journal of Corporate Accounting & Finance. 2008;19(2):25-31.

5.	Zhong N, Liu Z, He J, Su C. The structural problem of China’s non-financial corporate debt. Economic Research Journal. 2016;51(07):102-17.

6.	Du J, Li C, Wang Y. Shadow banking of non-financial firms: Arbitrage between formal and informal credit markets in China. Journal of Financial Intermediation. 2023;55:101032.

7.	Tian G. Influence of digital finance on household leverage ratio from the perspective of consumption effect and income effect. Sustainability. 2022;14(23):16271.

8.	Hadlock CJ, Pierce JR. New evidence on measuring financial constraints: Moving beyond the KZ index. The review of financial studies. 2010;23(5):1909-40.

9.	Fisher I. The debt-deflation theory of great depressions. Econometrica: Journal of the Econometric Society. 1933:337-57.

10.	Ahmad T, Zhang D, Huang C, Zhang H, Dai N, Song Y, et al. Artificial intelligence in sustainable energy industry: Status Quo, challenges and opportunities. Journal of Cleaner Production. 2021;289:125834.

11.	Liu Y, Xiong R, Lv S, Gao D. The impact of digital finance on green total factor energy efficiency: evidence at China’s city level. Energies. 2022;15(15):5455.

12.	Li Z, Chen H, Mo B. Can digital finance promote urban innovation? Evidence from China. Borsa Istanbul Review. 2023;23(2):285-96.

13.	Das S, Chatterjee A. Impacts of ICT and digital finance on poverty and income inequality: a sub-national study from India. Information Technology for Development. 2022:1-28.

14.	Ge H, Li B, Tang D, Xu H, Boamah V. Research on digital inclusive finance promoting the integration of rural three-industry. International Journal of Environmental Research and Public Health. 2022;19(6):3363.

15.	Bett F, Bogonko J. Relationship between digital finance technologies and profitability of banking industry in Kenya. International Academic Journal of Economics and Finance. 2017;2(3):34-56.

16.	Zhang P, Wang Y, Wang R, Wang T. Digital finance and corporate innovation: evidence from China. Applied Economics. 2023:1-24.

17.	Yuhui D, Zhang L. Regional digital finance and corporate financial risk: Based on chinese listed companies. Emerging Markets Finance and Trade. 2023;59(2):296-311.

18.	Ozili PK, Iorember PT. Financial stability and sustainable development. International Journal of Finance & Economics. 2023.

19.	Ozili PK. Impact of digital finance on financial inclusion and stability. Borsa Istanbul Review. 2018;18(4):329-40.

20.	Houston JF, Shan H. Corporate ESG profiles and banking relationships. The Review of Financial Studies. 2022;35(7):3373-417.

21.	Risman A, Mulyana B, Silvatika B, Sulaeman A. The effect of digital finance on financial stability. Management Science Letters. 2021;11(7):1979-84.

22.	Wang Q, Yang J, Chiu Yh, Lin TY. The impact of digital finance on financial efficiency. Managerial and Decision Economics. 2020;41(7):1225-36.

23.	Sun G, Li T, Ai Y, Li Q. Digital finance and corporate financial fraud. International Review of Financial Analysis. 2023;87:102566.

24.	Cui L, Wang Y. Can corporate digital transformation alleviate financial distress? Finance Research Letters. 2023:103983.

25.	Mu W, Liu K, Tao Y, Ye Y. Digital finance and corporate ESG. Finance Research Letters. 2023;51:103426.

26.	Razzaq A, Yang X. Digital finance and green growth in China: Appraising inclusive digital finance using web crawler technology and big data. Technological Forecasting and Social Change. 2023;188:122262.

27.	Yuan F. Research on the Impact Evaluation of Digital Finance on the Synergy between Economic Development and Ecological Environment. Journal of Environmental and public health. 2022;2022.

28.	Li J, Wu Y, Xiao JJ. The impact of digital finance on household consumption: Evidence from China. Economic Modelling. 2020;86:317-26.

29.	Jiang Z, Ma G, Zhu W. Research on the impact of digital finance on the innovation performance of enterprises. European Journal of Innovation Management. 2022;25(6):804-20.

30.	Wu Y, Huang S. The effects of digital finance and financial constraint on financial performance: firm-level evidence from China's new energy enterprises. Energy Economics. 2022;112:106158.

31.	Chang K, Cheng X, Wang Y, Liu Q, Hu J. The impacts of ESG performance and digital finance on corporate financing efficiency in China. Applied Economics Letters. 2023;30(4):516-23.

32.	Dai D, Fu M, Ye L, Shao W. Can Digital Inclusive Finance Help Small-and Medium-Sized Enterprises Deleverage in China? Sustainability. 2023;15(8):6625.

33.	Liu J, Jiang Y, Gan S, Chen R. The impact of D&O insurance on excess corporate leverage. China Journal of Accounting Research. 2022:100271.

34.	Baker ED, Boulton TJ, Braga-Alves MV, Morey MR. ESG government risk and international IPO underpricing. Journal of Corporate Finance. 2021;67:101913.

35.	Abbana S, Marimuthu F. Financing of state-owned entities: can the trade-off theory explain the debt structure? International Journal of Entrepreneurship and Sustainability Studies; Vol 3, Issue 1. 2023.

36.	Wang J, Gochoco‐Bautista MS, Sotocinal NR. Corporate investments in Asian emerging markets: Financial conditions, financial development, and financial constraints. Asian Development Bank Economics Working Paper Series. 2013;(346).

37.	Nyeadi JD, Sare YA, Aawaar G. Determinants of working capital requirement in listed firms: Empirical evidence using a dynamic system GMM. Cogent Economics & Finance. 2018;6(1):1558713.

38.	Dalci I, Ozyapici H. Working capital management policy in health care: The effect of leverage. Health Policy. 2018;122(11):1266-72.

39.	Chiou J-R, Cheng L, Wu H-W. The determinants of working capital management. Journal of American Academy of business. 2006;10(1):149-55.

40.	Luo S. Digital finance development and the digital transformation of enterprises: based on the perspective of financing constraint and innovation drive. Journal of Mathematics. 2022;2022:1-10.

41.	Kong D, Liu B. Digital technology and corporate social responsibility: evidence from China. Emerging Markets Finance and Trade. 2023:1-27.

42.	Guo F, Wang J, Wang F, Kong T, Zhang X, Cheng Z. Measuring the development of digital inclusive finance in China: Index compilation and spatial characteristics. China Econ Q. 2020;19(4):1401-18.

43.	Chen Z, Gao K, Huang W. Stock liquidity and excess leverage. Finance Research Letters. 2020;32:101178. doi: <https://doi.org/10.1016/j.frl.2019.04.034>.

44.	Zhang C, Zhu Y, Zhang L. Effect of digital inclusive finance on common prosperity and the underlying mechanisms. International Review of Financial Analysis. 2024;91:102940.

45.	Hu G, Wang S, editors. Digital financial inclusion, Financial Mismatch and Small and medium-sized enterprises Financing Constraints. SHS Web of Conferences; 2023: EDP Sciences.

46.	Buckley RP, Arner D, Zetzsche DA, Veidt R. Sustainability, FinTech and financial inclusion. A Research Agenda for Financial Inclusion and Microfinance. 2019.

47.	Ganguly S, Harreis H, Margolis B, Rowshankish K. Digital risk: Transforming risk management for the 2020 s. McKinsey & Company. 2017.
